# Telemedicine: a technological revolution

**DOI:** 10.1590/S1516-31802012000500001

**Published:** 2012-11-13

**Authors:** Alessandro Wasum Mariani, Paulo Manuel Pêgo-Fernandes

**Affiliations:** I MD. Thoracic Surgeon, Instituto Dante Pazzanese de Cardiologia, and Postgraduate Student in the Discipline of Thoracic and Cardiovascular Surgery, Faculdade de Medicina da Universidade de São Paulo (FMUSP), São Paulo, Brazil.; II MD, PhD. Associate Professor, Discipline of Thoracic Surgery, Instituto do Coração (InCor), Hospital das Clínicas (HC), Faculdade de Medicina da Universidade de São Paulo (FMUSP), São Paulo, Brazil.

Telemedicine and telehealth are terms that encompass the use of communications technology for the purposes of healthcare, which might be in relation to dissemination of knowledge, or in relation to patient care at a remote consultation office by a specialist, and so on. This segment of medicine already has a history of around 40 years of evolution, and thus is no longer so new, yet it is regarded with mistrust by many professionals. Nevertheless, telemedicine is clearly growing in a sustained manner. Initiatives and programs based on telemedicine are increasingly present in modern medical practice.

The United States is not only the birthplace of telemedicine but also a country in which its practice is firmly consolidated. Some examples of its application within American medicine include: remote monitoring of vital signs; transmission of images for interpretation and production of reports on radiological, anatomopathological and cardiological examinations, among others; patient consultations via teleconferencing; continuing medical education; information portals aimed towards patients; quick-consultation medical information applications for wireless devices; remote advice from specialist physicians to general practitioners in contact with patients; and data-gathering for clinical research; among many other uses. The American Telemedicine Association is a not-for-profit association founded in 1993 that has the aim of developing and organizing telemedicine within the United States. It has listed the following benefits from using telemedicine:^1^

1. Ease of access: through telemedicine, high-value medical information can even reach and be obtained from remote areas, without the need for patients or professional teams to move from their locations;
2. Cost reduction: as well as the reduced need for transportation and decreased length of hospital stay, the greater efficiency of treatment attained gives rise to better use of resources with less waste;
3. Greater convenience for patients: reduction of the need to travel and the rapid reaction of the medical team to any appearance of change produce greater satisfaction among patients and members of their families.

A good perspective on the growth within this field can be obtained by searching for the terms telemedicine and telehealth in the PubMed database, which yields 14,748 items, among which the earliest is dated 1974. It can be seen that the number of published papers on this topic per year remained small until the 1990s, when this rate shot up from just a few to hundreds of studies. This increasing trend continued through the 2000s to reach a peak in 2011, with a total of 1,224 indexed published papers. Many studies have proven that good results are achieved through using telemedicine in its various aspects. In 2012, Weaver and Murdock demonstrated the value of telemedicine for primary screening for retinopathy associated with prematurity. In a study evaluating a total of 582 examinations, all performed remotely, on a population of 137 premature neonates, these authors found a diagnosis of retinopathy in 13 infants.^2^ A mobile phone-linked telemedicine system for monitoring cardiac arrhythmia was used by Kirtava et al., with results published in 2012. Thirty-five patients with arrhythmia and seven healthy volunteers were assessed by means of a telecardiological device, which was shown to be efficient for diagnosing heart rate alterations, not only in three of the patients but also, surprisingly, with a diagnosis of arrhythmia in one of the healthy volunteers. This study serves to demonstrate the viability and efficiency of telemedicine for monitoring and making an early diagnosis of arrhythmias among outpatients.^3^

Among the many studies on the efficacy of using tele-education within medicine, Pereira et al. recently published a successful experience achieved in Brazil. These authors examined the teleconferencing program of Hospital das Clínicas, Universidade Estadual de Campinas, for communication with other institutions (both in Brazil and abroad) between September 2009 and August 2010. The evaluation demonstrated that telemedicine was a valuable tool for education, since it promoted interchange of knowledge, exchange of experiences, discussions and development of research within the field.^4^

In this regard, in Brazil, telemedicine has developed with strong links to distance medical education, and has been used by many authors as a synonym for tele-education within medicine. Several Brazilian universities now have groups dedicated to telemedicine with links to healthcare information technology. These initiatives are fundamental for the development of Brazilian medicine, both within each school and also for extramural dissemination of knowledge. The RUTE program (Rede Universitária de Telemedicina, i.e. University Telemedicine Network) is one example of how telemedicine can bring together centers of learning throughout the country, thereby significantly diminishing the inequalities between them.

Moreover, other aspects of telemedicine are starting to rise up in Brazil. Consultancy and diagnostic examination companies by tele-medicine (for example in relation to electrocardiography and radiology) are already functioning in this country. However, remote monitoring of vital signs is very little used in Brazil and still is very costly. Its use for directly attending patients is hindered by a lack of technical and ethical norms.

One important initiative is the “Programa Telessaúde Brasil Redes” (Brazilian Telehealth Network Program), created by the federal government in 2007. This project consists of integration of teaching and services through using information technology tools to promote tele-assistance and tele-education.^5^ The program aims to reach the following objectives:

1. Improvement of the quality of primary care attendance within the Brazilian National Health System (Sistema Único de Saúde, SUS);
2. Significant reduction of the cost of and time taken for personnel transportation;
3. Establishment of healthcare professionals in locations with difficult access;
4. Greater speed in the attendance provided;
5. Optimization of resources within the system as a whole, thus benefiting approximately 10 million SUS users.

Telemedicine represents a frontier within healthcare, but its development and use by the great majority of healthcare professionals is still well below its real potential. Nonetheless, its evolution is gathering pace and this healthcare segment is undoubtedly going to form an increasingly large part of our professional routines. In future editorials, we will be further discussing the various aspects of telemedicine.
